# Neutral theory reveals the challenge of bending the curve for the post‐2020 Global Biodiversity Framework

**DOI:** 10.1002/ece3.8097

**Published:** 2021-09-14

**Authors:** Falko T. Buschke

**Affiliations:** ^1^ Centre for Environmental Management University of the Free State Bloemfontein South Africa; ^2^ Animal Ecology, Global Change and Sustainable Development KU Leuven Leuven Belgium

**Keywords:** biodiversity indicators, Convention on Biological Diversity, counterfactuals, extinction, *Living Planet* *Index*, neutral theory, *Red List Index*

## Abstract

In October, nations of the world will begin negotiations for the post‐2020 Global Biodiversity Framework under the Convention on Biological Diversity. An influential ambition is “bending the curve of biodiversity loss,” which aims to reverse the decline of global biodiversity indicators. A second relevant, yet less prominent, milestone is the 20th anniversary of the publication of *The Unified Neutral Theory of Biodiversity and Biogeography*. Here, I apply neutral theory to show how global biodiversity indicators for population size (*Living Planet Index*) and extinction threat (*Red List Index*) decline under neutral ecological drift. This demonstrates that declining indicators are not solely caused by deterministic species‐specific or geographical patterns of biodiversity loss. Instead, indicators are sensitive to nondirectional stochasticity. Thus, “bending the curve” could be assessed relative to a counterfactual based on neutral theory, rather than static baselines. If used correctly, the 20‐year legacy of neutral theory can be extended to make a valuable contribution to the post‐2020 Global Biodiversity Framework.

## Graphical abstract

Global biodiversity indicators for populations (Living Planet Index) and extinction threat (Red List Index) decline under random ecological drift. The consequence of this is that prominent aspirations to “bend the curve of biodiversity loss” may be untenable, at least in terms of current biodiversity metrics. The Unified Neutral Theory of Biodiversity and Biogeography—which celebrates its 20th anniversary this year—could be used as a counterfactual against which to measure progress toward the post‐2020 Global Biodiversity Framework.

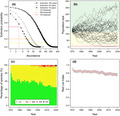

In October, nations of the world will meet in Kunming, China, where they will begin negotiating a post‐2020 Global Biodiversity Framework under the Convention on Biological Diversity. The overall failure to meet global biodiversity targets during the previous decade (IPBES, 2019; Secretariat of the Convention on Biological Diversity, 2020) has raised the stakes for these negotiations. One prominent ambition is “bending the curve of biodiversity loss” (Mace et al., 2018; Díaz et al., 2020; Leclère et al., 2020) because the next three decades should not only stop the downward trajectories of population sizes and extinction threats, but also redirect these trajectories upwards.

Although “bending the curve” is an engaging visual metaphor for the post‐2020 Global Biodiversity Framework, it needs to be made tractable by science‐based biodiversity targets and indicators. The global indicators being put forward to monitor the post‐2020 Global Biodiversity Framework necessarily reduce uncountable biological complexity into simple metrics (SBSTTA, 2020). These indicators include the *Living Planet Index,* a global indicator of vertebrate population trends (Collen et al., 2009; WWF, 2020); and the IUCN Red List (IUCN, 2012) and its associated *Red List Index* (Butchart et al., 2004), global indicators of incremental extinction threat. These indicators were integral to the global assessment of the Intergovernmental Science‐Policy Platform for Biodiversity and Ecosystem Services (IPBES, 2019), the Global Biodiversity Outlook Report 5 under the Convention on Biological Diversity (Secretariat of the Convention on Biological Diversity, 2020), and pioneering efforts to develop global pathways and mitigation scenarios for biodiversity (Leclère et al., 2020). These two indicators define the curve that needs to be bent by midcentury, and it is tempting to interpret downward or upward trends in these indicators as signs of human impact or conservation effectiveness, respectively. But is this interpretation always true?

It is not enough that indicators rise or fall with underlying biodiversity variables; upward or downward trends should also be attributable to *real* biological changes rather than random dynamics. This point is largely ignored in global biodiversity monitoring frameworks, which tend to interpret indicators relative to static baselines. However, as the Global Biodiversity Framework dominates our collective attention, this year also marks a second, less conspicuous, milestone: the 20th anniversary of the publication of *The United Neutral Theory of Biodiversity and Biogeography* (Hubbell, 2001). In the following sections, I posit that neutral theory provides valuable insights into the way global biodiversity indicators behave under random dynamics.

Neutral theory has established a controversial legacy over the last two decades by showing how simple stochastic births, deaths, speciation, and migration can predict many patterns in nature. The controversy stems from neutral theory's assumption that individual organisms are demographically equivalent (i.e., neutral), even though this assumption is obviously false (Hubbell, 2001; Leroi et al., 2020; Rosindell et al., 2011). Nevertheless, neutral theory answers the question of what biodiversity patterns would look like if individuals of different species were interchangeable. Often, such neutral patterns are indistinguishable from empirical data, much to the chagrin of those studying the nuanced natural histories of different species. Therefore, neutral theory could serve as a valuable null model for global biodiversity indicators.

Here, I will use the simplest possible model based on neutral theory to illustrate how global biodiversity indicators behave in the absence of any deterministic species‐specific threats. It would be convenient if these indicators were stable in the absence of deterministic species‐level trends, but, as I demonstrate in the subsequence sections, this is not the case. I specifically present the most basic neutral model for two reasons. First, I hope to portray neutral theory in a way that is accessible to nonspecialists, particularly the scientists and policymakers working toward the post‐2020 framework. Second, I want to demonstrate that even the coarsest neutral approximations can have heuristic value for global biodiversity policy.

The simple neutral model used here for illustrative purposes considers a saturated community of *J* = 5,000 individuals from *S = *40 species across 50 years between 1970 and 2020. The community is closed to migration and speciation rates are zero (although these processes can be included in more complex neutral models using the parameters *m* and *ν*, respectively: Hubbell, 2001). At the start of the simulation in 1970, the *J* individuals are randomly assigned to the *S* species. In every subsequent year, all individuals die and are replaced (i.e., zero‐sum dynamics), but the relative probability that a replacement belongs to a specific species is proportional to that species' relative abundance in the preceding year. In ecological terms, this could be imagined as a community of annual plants with nonoverlapping generations and without a long‐lived seedbank. Each year, all individual plants die after producing a fixed number of seeds regardless of their species identity, so that the structure of the plant community in the next year depends on how many seeds were produced the year before. As the years pass, the effect of random deaths and births accumulates so that some populations become more common, while others gradually decline. Such random fluctuations of species abundance are known as ecological drift (Hubbell, 2001).

The purpose of this model is not necessarily to replicate some real ecological community. The parameters for *J* and *S* are arbitrary, as is the decision to replace the whole community every year. What is important is that the fates of these species are completely random and that populations are equally likely to increase or decrease under ecological drift (Figure 1a). Moreover, the zero‐sum dynamics ensure that for every population that declines randomly, another increases in equal measure.

**FIGURE 1:**
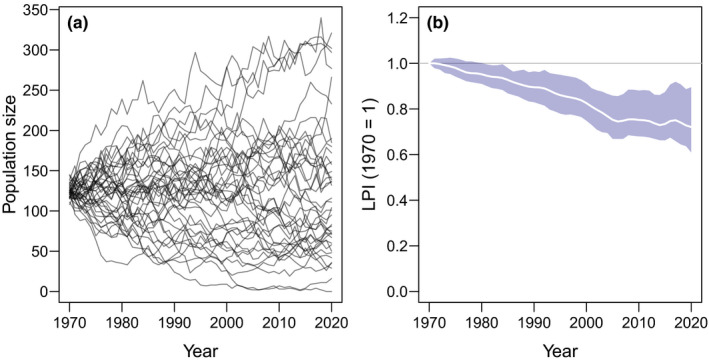
The Living Planet Index for a neutral community. (a) Zero‐sum ecological drift of *J* = 5,000 individuals across *S* = 40 species between 1970 and 2020. (b) The mean and 95% confidence intervals of *Living Planet Index* (LPI) for the neutral community, where the baseline is set to population abundance in 1970

Despite the growth or decline of populations being equally probably, random ecological drift results in a consistently declining *Living Planet Index* (Figure 1b). One expects the *Living Planet Index* would remain stable in the absence of deterministic trends, but ecological drift in this simple neutral model caused the index to decline by as much as 20% in 50 years, even though the total number of individual organisms remained unchanged (Figure 1). The *Living Planet Index* declines because it is based on year‐on‐year changes in populations measured as *λ* = log(*N_t_*_+1_/*N_t_*) (Collen et al., 2009; WWF, 2020). This formulation was designed for exponential population growth, where doubling a population is symmetrical to halving the same population, even though the absolute change in the population is twice as much in the former. Even though every declining population in the neutral model must be accompanied by an equivalent increasing population, the log‐transformation ensures that positive fluctuations cannot compensate for negative fluctuations, hence a declining index (Buschke et al., 2021). This asymmetry is largest in small populations due to the smaller denominator when calculating *λ* (Buschke et al., 2021).

The same neutral model can be used to explore indicators of extinction threat. In the absence of dispersal or speciation, all species in neutral communities will eventually drift to extinction, except for one random species that becomes mono‐dominant, so neutral simulations can be iterated to estimate how species' abundances affect their extinction rates (Figure 2a). The IUCN Red List defines three levels of extinction threat (IUCN, 2012): *critically endangered* (CR: 50% extinction probability within 10 years), *endangered* (EN: 20% extinction probability within 20 years), or *vulnerable* (VU: 10% extinction probability within 100 years), so I allowed neutral populations to fluctuate randomly and calculated how long it took them to drift to extinction (Figure 2a). Using 10,000 iterations of the neutral model, I measured how often species went extinct across three time intervals (10, 20, and 100 years) for every increment of species abundance. This allowed me to quantify how ecological drift caused species to transition between Red List categories (Figure 2b) and then calculate the proportion of threatened species in the community through time (Figure 2c). The increasing frequency of threatened populations caused a declining *Red List Index* (Figure 2d).

**FIGURE 2:**
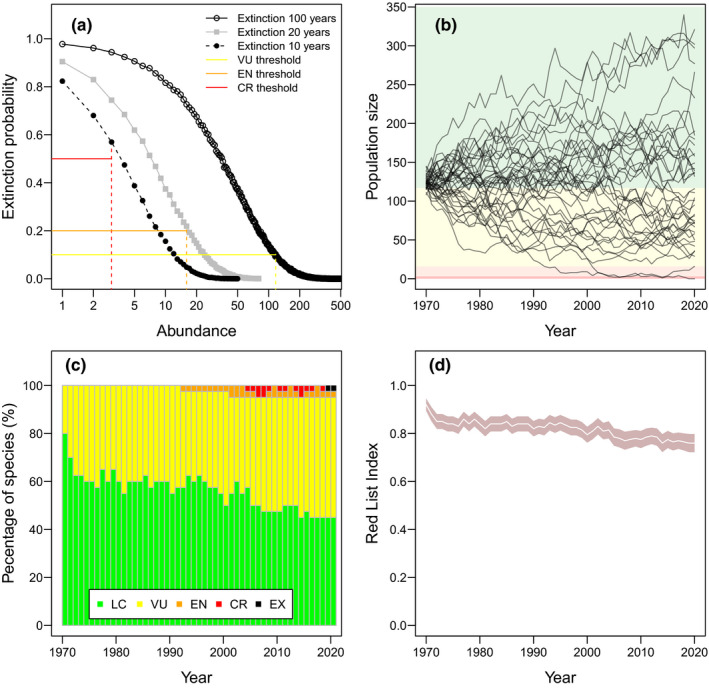
The extinction risk of a neutral community. (a) Extinction probability as a function of abundance across three time intervals (10, 20, and 100 years) for a neutral community of *J* = 5,000. Colored lines show the thresholds for critically endangered (CR), endangered (EN), and vulnerable (VU) populations. (b) The zero‐sum ecological drift of *J* = 5,000 individuals from *S* = 40 species between 1970 and 2020, superimposed with colored thresholds of extinction threat categories. (c) The percentage of each species per threat category between 1970 and 2020, and (d) the associated *Red List Index* (where values of 1 indicate all species are Least Concern, LC, and 0 indicates that all species are extinct, EX)

Ecological drift causes the *Red List Index* to decline because the thresholds between threat categories do not scale linearly (i.e., the log‐scale of the x‐axis in Figure 2a). Random population declines, especially in smaller populations, are more likely to cross thresholds between threat categories than equivalent random increases in larger populations, so randomly improving threat statuses are unable to compensate for randomly worsening threat statuses. Inevitably, ecological drift leads to a declining *Red List Index*. This adds context to calls for defining a headline global conservation priority based on extinction (Rounsevell et al., 2020), because it implies that indicators of extinction risk gradually worsen due to random chance alone (Figure 2d).

Declining biodiversity indicators will have policy ramifications if conservation scientists attribute these patterns to human pressures. The simple neutral model presented here shows how the *Living Planet Index* and the *Red List Index* can decline even in the absence of threatening processes or disproportionate sensitivity of certain species. This is significant considering that Goal A2 in the first draft of the post‐2020 Global Biodiversity Framework, proposes that “*The increase in the extinction rate is halted or reversed, and the extinction risk is reduced by at least 10 per cent, with a decrease in the proportion of species that are threatened, and the abundance and distribution of populations of species is enhanced or at least maintained*.” (Open‐ended Working Group on the post‐2020 Global Biodiversity Framework 2021). This goal unambiguously calls for an increasing *Living Planet Index* and *Red List Index,* trends that are unlikely under neutral ecological dynamics. Therefore, deterministic trajectories in biodiversity indicators should be assessed relative to declines caused by random ecological dynamics.

By this point, critics will be crying out that biodiversity loss is obviously non‐neutral. There is considerable evidence that population declines, and extinction risks vary across taxonomic groups and biogeographical regions (Hilbers et al., 2017; Leung et al., 2020; WWF, 2020). However, the purpose of neutral theory here is not to explain the underlying cause of biodiversity patterns, but rather to predict what patterns would look like if species were equivalent (Box 1). Neutral theory can be used as a counterfactual by modelling multiple biodiversity targets simultaneously even in the absence of species‐ or threat‐specific empirical data. Thus, it can contribute to agenda‐setting and target formulation or applied retrospectively during target review (*cfr*. Nicholson et al., 2019). Furthermore, even though the model presented here was purposely simplistic, simulations can be made more sophisticated by adding dispersal and speciation. Adjusting these parameters have, for example, already been used to predict extinction debt following habitat fragmentation (Thompson et al., 2019) or whether restoration can mitigate human impacts (Buschke & Sinclair, 2019).

BOX 1 **If neutral theory is the answer, what is the question?**

Much of the debate around neutral theory stems from its inconsistent application and interpretation. Neutrality does not fully explain the mechanisms that underlie biodiversity patterns. Even proponents of neutral theory accept that the world is distinctly non‐neutral (e.g., Hubbell, 2001; Leroi et al., 2020; Rosindell et al., 2012). Instead, neutral theory can either be used as a null model or as a predictive approximation, but not both, depending on its fit to empirical data. For example, where neutral predictions fail to match empirical data, neutral theory serves as a null model by showcasing deterministic effects. In such instances, it is necessary to state informative alternative hypotheses for the empirical patterns (Gotelli & McGill, 2006; McGill et al., 2006). By contrast, neutral predictions that match empirical data are not evidence for demographic equivalence, rather evidence that the empirical data are insufficient to detect any underlying determinism. When this is the case, neutral theory can be used as an efficient theory to predict biodiversity patterns without the need to parameterize more complex models (Marquet et al., 2014).

Given this, how should we interpret neutral theory in the context of global biodiversity indicators? In the main text, I showed how both the *Living Planet Index* (Figure 1) and the *Red List Index* (Figure 2) decline under neutral dynamics. Yet, this simple observation can inform vastly different interpretations, some more appropriate than others. Here are three illustrative interpretations of varying correctness:

1. **False:** *declines in biodiversity indicators are the result of random ecological drift*. This interpretation fails to treat neutral theory as a null model or as a prediction. Even if neutral predictions match empirical declines closely, this is not evidence that these declines are the consequence of random ecological drift. Instead, it simply means that ecological drift cannot be ruled out as one possible contributor to declining indicators.
2. **Partially true:** *neutral theory predicts the future trajectories of global biodiversity indicators*. Neutral theory can make accurate predictions, but only on condition that deterministic processes (e.g., species‐specific threats, or geographically implicit conservation) are too weak to overshadow the statistical effects of random births, deaths, and migration. When this is the case, neutral theory can be used for prediction.
3. **True:** *global biodiversity indicators are sensitive to neutral ecological dynamics*. This statement is plainly true based on our simple simulations because it treats neutral theory as a null model. Of consequence here is the rejection of the alternative hypothesis that indicators, such as the *Living Planet Index* and the *Red List Index*, are stable in the absence of deterministic upward or downward trends.

Therefore, as it is presented here, neutral theory answers questions about the statistical behavior of the indicators themselves, rather than the biodiversity patterns they aim to quantify. This is an important distinction that should be acknowledged to avoid drawing spurious conclusions from simple simulations.

If used correctly, the 20‐year legacy of neutral theory can be extended to make a valuable contribution to the post‐2020 Global Biodiversity Framework. This contribution will not be about proving that empirical biodiversity trends are due to random chance alone. Instead, neutral theory could be used to model counterfactuals against which empirical trends can be compared (Nicholson et al., 2019). Comparing empirical patterns to neutral simulations will allow us to pinpoint whether sensitive species contribute disproportionately to indicator declines or whether declines in one geographical region consistently differ from those in another region (e.g., Leung et al., 2020), given natural differences in species richness and abundance. Therefore, neutral theory provides a deeper understanding of the sensitivity of global biodiversity indicators to ecological stochasticity and in process helps us measure progress toward “bending the curve of biodiversity loss” more accurately.

## CONFLICT OF INTEREST

None declared.

## AUTHOR CONTRIBUTIONS

**Falko T. Buschke:** Conceptualization (lead); data curation (lead); formal analysis (lead); investigation (lead); methodology (lead); project administration (lead); visualization (lead); writing‐original draft (lead); writing‐review & editing (lead).

## Data Availability

All codes used in the submission are available from Zenodo (https://doi.org/10.5281/zenodo.5118552).
